# Sustainable biosynthesis, physiochemical characterization, cytotoxicity, and antimicrobial evaluation of novel chromium oxide nanoparticles

**DOI:** 10.3389/fchem.2025.1584199

**Published:** 2025-09-24

**Authors:** Ezzat H. Elshazly, G. Abd elfadeel, Lihang Yang, Xiqi Li, Emad A. Ewais, Ahmed M. Sadek, Taher M. Taha, Omar Fathy, Omar Mohammad Atta, Wen-Zong Liu

**Affiliations:** 1 Shenzhen Key Laboratory of Organic Pollution Prevention and Control, School of Eco-Environment, Harbin Institute of Technology Shenzhen, Shenzhen, China; 2 Department of Botany and Microbiology, College of Science, Al-Azhar University, Assiut, Egypt; 3 Physics Department, Faculty of Science, Al-Azhar University, Assiut, Egypt; 4 Botany and Microbiology Department, Faculty of Science, Al-Azhar University, Cairo, Egypt; 5 State Key Laboratory of Urban Water Resource and Environment, Harbin Institute of Technology (SKLUWRE, HIT), Harbin, China

**Keywords:** structure properties, optical properties, cytotoxicity, antimicrobial, physical activity

## Abstract

The biosynthesis of nanoparticles (NPs) has attracted significant interest due to their diverse biological applications. However, the potential for NPs synthesis using plant resources from *Vicia monantha* Retz remains largely unexplored. Notably, this study marks the first use of this specific plant for the biosynthesis of chromium oxide nanoparticles (Cr_2_O_3_NPs). In the present study, the single phase of Cr_2_O_3_ was confirmed at a calcination temperature 700 °C for the synthesized NPs. The crystallite sizes increased from 14 nm to 20 nm with the increase in the calcination temperature to 900 °C for 2 h. Ultraviolet–visible (UV–VIS) light spectroscopy revealed that the samples are semiconductor materials, according to the observed values of energy band gap. The developed Cr_2_O_3_NPs did not show any toxicity toward NIH-3T3 fibroblasts. The results demonstrated that Cr_2_O_3_NPs exhibited good antimicrobial activity against two bacterial strains (*Escherichia coli* and *Staphylococcus aureus*) and two fungal strains (*Candida albicans* and *Aspergillus sp*.), producing clear inhibition zones of 0.26 cm, 0.21 cm, 0.28 cm, and 0.3 cm, respectively, after 24 h. The Cr_2_O_3_NPs exhibit successful green synthesis, notable biocompatibility, and antimicrobial properties, making them highly promising for various applications and opening possibilities for the utilization of nanoparticles in antimicrobial systems.

## 1 Introduction

Bio-nanotechnology has become a significant area within nanotechnology because of its environmental sustainability, simplicity, and economic efficiency (Shahcheraghi et al., 2022). The combination of metallic and botanical structures creates an effective basis for the production of nanoparticles (NPs) with a variety of properties (Sawalha et al., 2021). Nanoparticles have been used in a variety of fields, including cosmetics, sensors, biomedicine, pathogen detection, diagnostics, antigen detection, enzymes, vaccines, and radiology, because of their high-density corners, chemical stability, and exceptional magnetic, catalytic, and electrical properties (Iqbal et al., 2020a). Nanoparticles such as zinc oxide, silicon dioxide, titanium dioxide, silver, and carbon nanotubes are harmful to the environment and humans. These NPs can cause cell death, oxidative stress, DNA damage, apoptosis, and inflammation (Sengul and Asmatulu, 2020; Srivastava et al., 2015). These issues necessitate safer nanoparticles. Chromium oxide nanoparticles (Cr_2_O_3_NPs) offer a promising alternative due to their unique physicochemical properties. Cr_2_O_3_ NPs are unique among metal oxide nanoparticles due to their extraordinary stability, hardness, high resistivity, higher melting temperature, and 3.4 eV band gap (Iqbal et al., 2020b). Nanoparticles are characterized by a high surface area to volume ratio, which is a contributing factor to their distinctive physicochemical properties (Joudeh and Linke, 2022). This surface area also enhances their antimicrobial effectiveness and reactivity, rendering them suitable for use in medical devices and antimicrobial coatings (Rezić and Meštrović, 2023). Cr_2_O_3_NPs have several uses, including catalysis, photonics, coatings, and complex colorants (Karimian and Piri, 2013). The most stable of the chromium oxides are the trivalent Cr_2_O_3_NPs (Horie et al., 2013). Despite their promise, Cr_2_O_3_NPs have limited biological uses due to reported harmful effects in several studies (Alqarni et al., 2022).

Antimicrobial nanoparticles, including silver and copper, demonstrate significant bactericidal efficacy (Fan et al., 2021). Nonetheless, these nanoparticles present cytotoxic risks to cells (Fan et al., 2021; Tortella et al., 2022). Chromium oxide nanoparticles demonstrate the ability to improve antimicrobial properties while reducing cytotoxic effects. Cr_2_O_3_ nanoparticles compromise bacterial membranes and impede enzyme function, resulting in significant antimicrobial efficacy (Khan et al., 2021). The biocompatibility of Cr_2_O_3_NPs is critical for their application in a variety of biological systems. Coating or functionalizing the surfaces of Cr_2_O_3_NPs with biogenic compounds might mitigate their negative effects. A possible strategy is to cover the surface of Cr_2_O_3_NPs with biogenic phytomolecules derived from plants (Khan et al., 2021). Thus, green nanoparticle manufacturing has emerged as a viable alternative to traditional physical and chemical approaches, with the potential to mitigate some of their negative impacts (Ahmad et al., 2022; Khan et al., 2021). Green synthesis is an interesting method for manufacturing nanoparticles since it is straightforward, inexpensive, and ecologically benign (Khan et al., 2021). In cancer therapy, biologically synthesized metallic nanoparticles function as cytotoxic agents (Patil and Chandrasekaran, 2020). Plant extracts, unlike bacteria and fungi, offer a straightforward and effective method for producing large-scale nanoparticles (Singh et al., 2018).

Numerous researchers are currently focusing on the green manufacture of Cr_2_O_3_NPs using plant extracts (Zheng et al., 2021). Plant extracts include a variety of phytochemicals, including phenols, flavonoids, and terpenoids, which possess natural reducing and stabilizing properties. These compounds can effectively convert metal salts into nanoparticles under mild reaction conditions, reducing the need for hazardous chemicals and high-energy procedures. This approach produces nanoparticles with a precise size distribution and remarkable stability (Adeyemi et al., 2022). Because of its environmentally friendly methodology and broad applicability, green nanoparticle production utilizing plant extracts has a lot of potential in nanotechnology (Khan et al., 2021). Cr_2_O_3_NPs have significant antibacterial activity. Their ability to suppress microbial development makes them ideal for medical applications such as medication delivery systems and medical device coatings. Research has demonstrated that these nanoparticles may successfully target a variety of pathogens, including bacteria and fungi, enhancing their potential for infection management (Adfar et al., 2023). Although further research is needed to completely understand the reasons and enhance nanoparticle-based treatments, these improvements provide intriguing possibilities for novel treatment options. *Vicia sativa L*. is an important crop often known as common or garden vetch. It is a grain legume with excellent seed yield, which may reach 250 kg per hectare. The seeds are high in protein, carbohydrates, and minerals, making them suitable for both human diet and animal feed (Aloweidat, 2014). Common vetch also has several pharmacological characteristics. *V. sativa* has lately been used as a fertilizer to increase soil nitrogen availability, and it may be cultivated all-year to provide green manure (Ramírez-Parra and De la Rosa, 2023). *V. sativa* is also known as a powerful medicinal plant due to its high concentration of flavonoids, phenolics, saponins, tannins, and terpenoids (Ahmed et al., 2019).

The current study comprehensively describes the reaction conditions, synthesis technique, and properties of Cr_2_O_3_NPs. Cr_2_O_3_NPs were first biosynthesized from chromium acetate, with an aqueous leaf extract of *V. sativa* serving as a reducing and stabilizing agent. Furthermore, Cr_2_O_3_NPs were characterized using a variety of approaches. In addition, numerous of its biological activities were studied, including cytotoxicity and antibacterial testing. The sustainable production of Cr_2_O_3_NPs offers a promising method for creating environmentally benign materials with important applications in catalysis, environmental remediation, and biomedicine. Characterization and assessment of their qualities are critical for realizing their full potential while maintaining safety and efficacy.

## 2 Empirical procedure

### 2.1 Preparation of the aqueous plant extract for nanoparticle synthesis

Two grams of the plant powder obtained from *Vicia monantha* was dissolved in 100 mL of deionized water and placed in an ultrasonic bath at 80 °C for 40 min. The solid material was then removed and filtered twice using Whatman filters to eliminate any residual solids. The filtrate was stored at 25 °C until it was used for the biosynthesis of Cr_2_O_3_NPs.

### 2.2 Synthesis of chromium nanoparticles

Cr_2_O_3_NPs were synthesized using chromium chloride (CrCl_3_) as a precursor. A mixture of the plant extract and 0.2 M CrCl_3_ solution (3:7 ratio) was stirred magnetically at 100 °C for 1.5 h in a 250-mL conical flask. Nanoparticle formation was monitored *via* color change. After cooling, Cr_2_O_3_NPs were separated by centrifugation (13,500 rpm at 4 °C) and dried at 80 °C. The nanoparticle concentration was determined using ultraviolet–visible (UV–VIS) spectrophotometry (Zainab et al., 2022). The prepared sample was ground completely using a mortar and pestle to obtain a fine powder. The powdered sample was divided into different parts to be calcined at different calcination temperatures (500 °C, 700 °C, and 900 °C).

### 2.3 Instrumentations and measurements

The X-ray diffraction (XRD) patterns of the as-prepared and calcined samples were obtained using an X-ray diffractometer (Philips model pw1710, Cu-Kα radiation, and λ = 1.54 Å) in the range of 10–80̊ (step 0.02) for 2θ. The average crystallite size (D) of Cr_2_O_3_ was calculated using Scherrer’s equation (Abd elfadeel et al., 2022):

$$D=\frac{k\;\lambda }{\beta_{hkl}\mathrm{Cos}\theta },$$ (1)

where *k,* β_hkl_, λ, and θ are the shape factor (0.9), the full width at half of the maximum intensity of the diffraction peak in radian, the wavelength of the used X-ray beam, and Bragg’s refractive angle, respectively.

Specific surface area (SSA), which refers to the area per unit mass, is considered an important factor to present the bulk rates of such reactions (m^2^/g). It is a significant parameter for nanoparticles because their large surface-to-volume ratio decreases with an increase in particle size. It can be calculated using the following formula (Al-Saadi and Hameed, 2015; Theivasanthi and Alagar, 2011; 2012):

$$SSA=\frac{6*10^{3}}{D_{p}\rho },$$ (2)

where ρ presents the density of Cr_2_O_3_NPs (5.23 g cm^-3^).

Fourier-transform infrared (FT-IR) spectra were recorded in the range of 400 cm^-1^–4,000 cm^-1^ using a double-beam spectrometer (Nicolet iS 10) with the KBr pellet method. The optical properties were determined using a computerized double-beam UV-Vis spectrophotometer (JASCO V-670). The absorbance spectrum of Cr_2_O_3_, both as prepared and calcined at different temperatures (500 °C, 700 °C, and 900 °C for 2 h) were recorded in the range of 200 nm–800 nm at room temperature. The energy band gap was calculated using Tauc’s formula (Venkatachalapathy et al., 2023):

$$\alpha h\nu ={A\left(h\nu -E_{g}\right)}^{0.5},$$ (3)

where α, h, ν, A, and E_g_ are the absorbance constant, Planck constant, the frequency of the incident photons, the transition constant, and the energy band gap, respectively (Venkatachalapathy et al., 2023).

### 2.4 Antibacterial activity

The antibacterial activity of Cr_2_O_3_NPs was evaluated using the disc diffusion method, as previously reported, against *Escherichia coli*, *Staphylococcus aureus*, *Candida albicans*, and *Aspergillus sp*. (Atta et al., 2021). In brief, nutrient agar medium or yeast–peptone–dextrose–agar (YPDA) medium was used to cultivate all the microbial strains. Cr_2_O_3_NP suspensions were applied to the 8-mm-diameter paper discs and then UV-sterilized. Following that, the dried discs were placed on top of the agar culture plates containing a specific set of bacteria and fungi that had been incubated for 24 h at 37 °C and 72 h at 30 °C, respectively. Finally, the inhibition zones’ diameters were measured. The negative and positive controls were dimethyl sulfoxide (DMSO) and chromium nitrate, respectively.

### 2.5 Toxicity analysis

The cytotoxicity of Cr_2_O_3_NPs on NIH-3T3 (mouse embryonic fibroblast cells obtained from China Infrastructure of Cell Line Resources) (Adhikari et al., 2024; Atta et al., 2021; 2022) was assessed using the MTT assay. NIH-3T3 cells were cultured in high-glucose (4.5 g/L) DMEM flasks supplemented with L-glutamine, pyruvate (110 g/L), 10% FBS (Gibco, United States), and 1% penicillin/streptomycin and incubated at 37 °C in a 5% CO_2_ atmosphere. The culture medium was replaced daily until confluence was achieved. For toxicity assessment, the Cr_2_O_3_NPs were positioned in a 96-well microplate, inoculated with 1 × 10^4 cells per well, and incubated in a 5% CO_2_ environment. Following 24 h–48-h of incubation, the samples were washed thrice with PBS and subsequently placed into fresh DMEM growth medium containing MTT (3-(4,5-dimethyl-2-thiazolyl)-2,5-diphenyl-2H tetrazolium bromide) reagent at a concentration of 5 mg/mL in a 10:1 ratio. The samples were re-incubated at 37 °C for 4 h. Subsequently, the medium was eliminated, and formazan, along with 150 mL of DMSO, was introduced. Absorption was ultimately quantified at 570 nm utilizing a multi-scan spectrophotometer (Tecan, Infinite F50, Germany). The cells cultured solely in the medium served as a reference, and their viability was regarded as 100% (Atta et al., 2022).

## 3 Results and discussion

### 3.1 Physicochemical investigations

The extract of *Vicia monantha* is essential in nanoparticle synthesis because its active compounds stabilize the nanoparticles, inhibit agglomeration, and improve crystallization, as demonstrated in a previous study (Nasr et al., 2024). The combination of metallic components and plant extracts improves the product’s functional and environmental performance, offering benefits such as biocompatibility, material stability, and increased metal strength (Jeevanandam et al., 2022). Plant-based materials have biodegradable and eco-friendly properties, which improve sustainability (Hano and Abbasi, 2021). Although pure metallic systems may provide greater durability, they lack the inherent benefits of plant extracts (Dikshit et al., 2021). Green synthesis improves the properties of Cr_2_O_3_ nanoparticles (Yasmeen et al., 2023). Our findings show that the plant extract significantly influences particle agglomeration and crystallization, thereby improving the overall stability and functionality of the nanoparticles.

**FIGURE 1:**
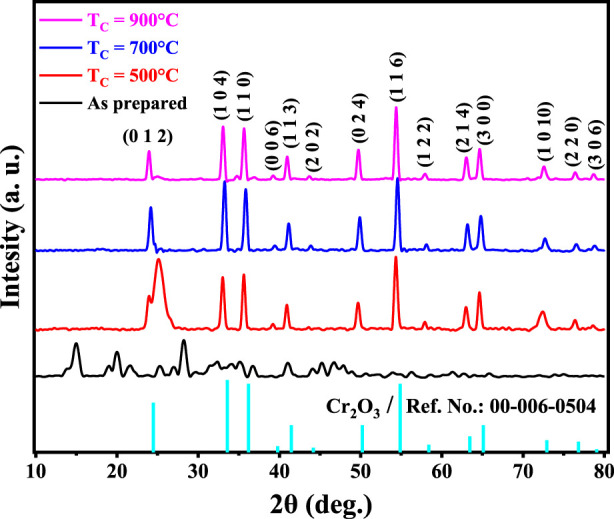
XRD patterns of Cr_2_O_3_ as prepared and calcined at different temperatures.

The XRD patterns of Cr_2_O_3_ as prepared and calcined at different temperatures (500 °C, 700 °C, and 900 °C for 2 h) are shown in Figure 1. The broad peaks observed in the XRD analysis of the material synthesized and calcined at 500 °C indicate a low degree of crystallinity. The crystallinity of the calcined materials showed an improvement with an increase in T_c_ (Abdel-Ghany et al., 2012; Abd elfadeel et al., 2023). The observed narrow XRD peaks with higher intensities were achieved as a function of T_c_. This means that the maximum crystallinity percentage can be obtained at a calcination temperature in the range of 700 °C ≤ T_c_ ≤ 900 °C. The XRD spectrum displayed distinct and intense peaks, confirming the crystalline nature and single-phase formation of Cr_2_O_3_NPs. The peaks of the XRD spectrum of the samples at calcination temperatures of 700 °C and 900 °C showed excellent agreement with those mentioned in previous works for Cr_2_O_3_ (Hao et al., 2019; Sackey et al., 2021).

Figure 1 clearly shows that the XRD pattern is dominated by diffraction peaks 0 1 2, 1 0 4, 1 1 0, 0 0 6, 1 1 3, 2 0 2, 0 2 4, 1 1 6, 1 2 2, 2 1 4, 3 0 0, 1 0 10, 2 2 0, and 3 0 6 that correspond to 2θ at 24.22°, 33.33°, 35.92°, 39.42°, 41.20°, 43.82°, 49.92°, 54.57°, 58.13°, 63.14°, 64.79°, 72.66°, 76.57°, and 79.04°, respectively. The noticeably strong and narrow diffraction peaks in the pattern of XRD are ascribed to the elevated crystallinity of the Cr_2_O_3_NPs. It is completely indexed in the space group R $\bar{3}$ c symmetry of the rhombohedral structure. No impurity peaks were detected, which is consistent with the reference value of JCP No. 00-006-0504.

The crystallite size increased from 14 nm to 20 nm as the calcination temperature was increased to 900 °C for 2 h. The increase in crystallite size is attributed to the growth of the magnetic domain as a function of the calcination temperature T_c_ for all samples (Mazen et al., 2015). It is noted that the obtained small crystallite size for the pure phase of α-CrO_3_ (14 nm–20 nm) is smaller than that of samples prepared by the microwave irradiation method (Meenambika et al., 2013).

Specific surface area values of Cr_2_O_3_NPs are presented in Table 1. SSA decreases slightly as the particle size and calcination temperature increase, which can be attributed to the generation of agglomeration as a result of the heat treatment occurring in the material (Al-Saadi and Hameed, 2015; Theivasanthi and Alagar, 2011; 2012).

**TABLE 1:** Crystallite size (D), optical absorption edge, SSA, and band gap (E_g_) of Cr_2_O_3_ powder nanoparticles at different calcination temperatures (T_c_).

Tc (°C)	As prepared	500 ℃	700 ℃	900 ℃
Crystal size D (nm)	14	19	20	20
SSA (m^2^.g^−1^)	78.5	58	54.2	54.2
Abs. edge (nm)	294	325	312	316
Eg (eV)	4.2	3.8	3.9	3.9

**FIGURE 2:**
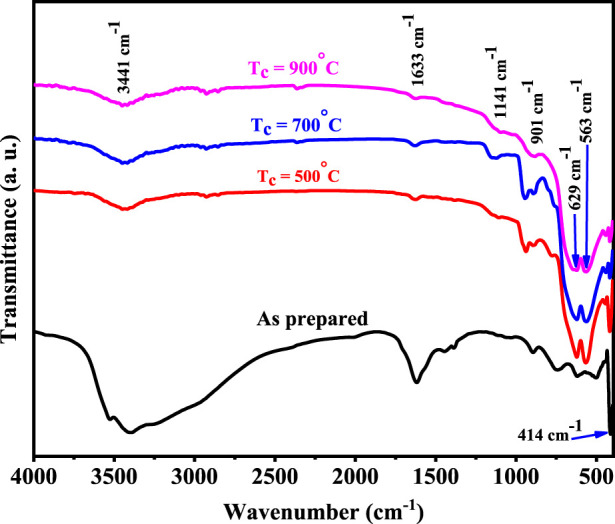
FT-IR spectra of Cr_2_O_3_ as prepared and calcined at different temperatures.

The FT-IR absorption spectra of Cr_2_O_3_ as prepared and calcined at different temperatures of 500 °C, 700 °C, and 900 °C for 2 h in the range of 4,000 cm^-1^–400 cm^-1^ are shown in Figure 2. Several vibrational modes were observed. The spectra represent absorption bands at 414, 451, 563, 629, 901, 952, 1,141, 1,633, and 3,441 cm^−1^, respectively. All the observed peaks are in good agreement with the previously reported results (Bumajdad et al., 2017; Ahmed Mohamed et al., 2020; Rasheed et al., 2020; Sone et al., 2016). The metal oxide generally represents the peaks caused by interatomic vibrations that are below 1,000 cm^−1^. So, in the present investigation, the vibrations positioned at 414, 451, 563, 629, 901, and 952 cm^−1^ can be attributed to the Cr–O stretching vibrations (Abdullah et al., 2014; Bumajdad et al., 2017; Hassan et al., 2019). The relatively weak absorption band at 414 cm^−1^ and the strong absorption band at 563 cm^−1^ can be attributed to Cr–O bonds in the bending mode, while the strong absorption band at 629 cm^−1^ provides clear evidence for the presence of crystalline α-Cr_2_O_3_ (Bumajdad et al., 2017; Jaswal et al., 2014; Sone et al., 2016). The relatively strong broad band observed at 3,441 cm^−1^ can be attributed to O–H stretching modes of what could be waters of hydration. The weak broad band at 1,633 cm^−1^ may be attributed to the presence of adsorbed moisture on the surface of the α-Cr_2_O_3_ powder (Adnan and Mohammed, 2024; Hassan et al., 2019; Jaswal et al., 2014; Ahmed Mohamed et al., 2020; Sone et al., 2016). The spectra of the calcination products (at 500 °C–900 °C) are significantly different from that of the as-prepared Cr_2_O_3_. The spectrum of Cr_2_O_3_ only exhibits two sharp, strong bands at 629 and 563 cm^−1^, in addition to a much weaker absorption at 901, 1,141, 1,633, and 3,441 cm^−1^ (Bumajdad et al., 2017). As the calcination temperature increases, it is observed that the broad peak groups associated with O–H stretching vibration shrink, while the stretching peaks of Cr_2_O_3_ grow and become significant. The spectrum of the calcined samples reveals the corresponding vibration bands of Cr–O only, which in turn depicts the successful removal of impurity during calcinations and the high purity of the as-grown Cr_2_O_3_ nanostructures. The high intensity of the peaks of the Cr_2_O_3_ bands indicates the good crystalline nature of the materials (Abdullah et al., 2014; Rasheed et al., 2020).

The values of crystallite size (D), optical absorption edge, and the band gap (E_g_) of the Cr_2_O_3_ powder nanoparticles at different calcination temperatures (Tc) are listed in Table 1.

**FIGURE 3:**
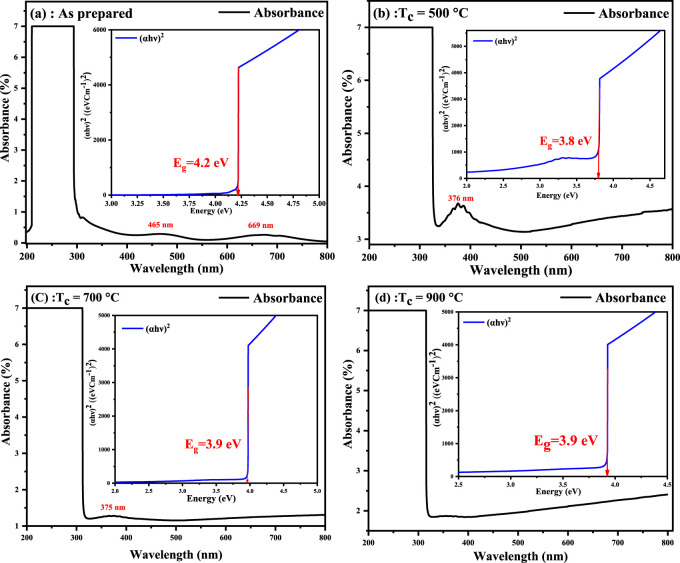
**(a–d)** UV–VIS absorption spectrum and the inserted Tauc’s plot for the band gap of as-prepared Cr_2_O_3_ and calcined Cr2O_3_ at different temperatures.

Sangwan and Kumar (2017) reported the optical characteristics of chromium oxide and observed that the energy gap of the electronic transitions is located in the photon energy range of 4.19 eV. The absorption wavelength of chromium oxide was noted in the visible light region, as shown in Figures 3a–d. The calculated values of the band gap were noted to be 4.2, 3.8, 3.9, and 3.9 eV, respectively. The values of the investigated absorption edges and the energy gap of Cr_2_O_3_NPs are in good agreement with the previous studies (Ahmad et al., 2018; Hassan et al., 2019; Kamble et al., 2019; Sangwan and Kumar 2017). It was observed that the value of the band gap decreased as the calcination temperature increased. The decrease in the optical band gap with increasing crystallinity confirms the quantum size effect, which is consistent with previous findings (Shobana et al., 2019). The peaks observed at approximately 375, 465, and 669 nm can be attributed to the 3-d electronic transitions of Cr^3+^ ions, which are situated in six-coordinate geometry with octahedral symmetry (Khalaji and Pavel Machek, 2022).

**FIGURE 4:**
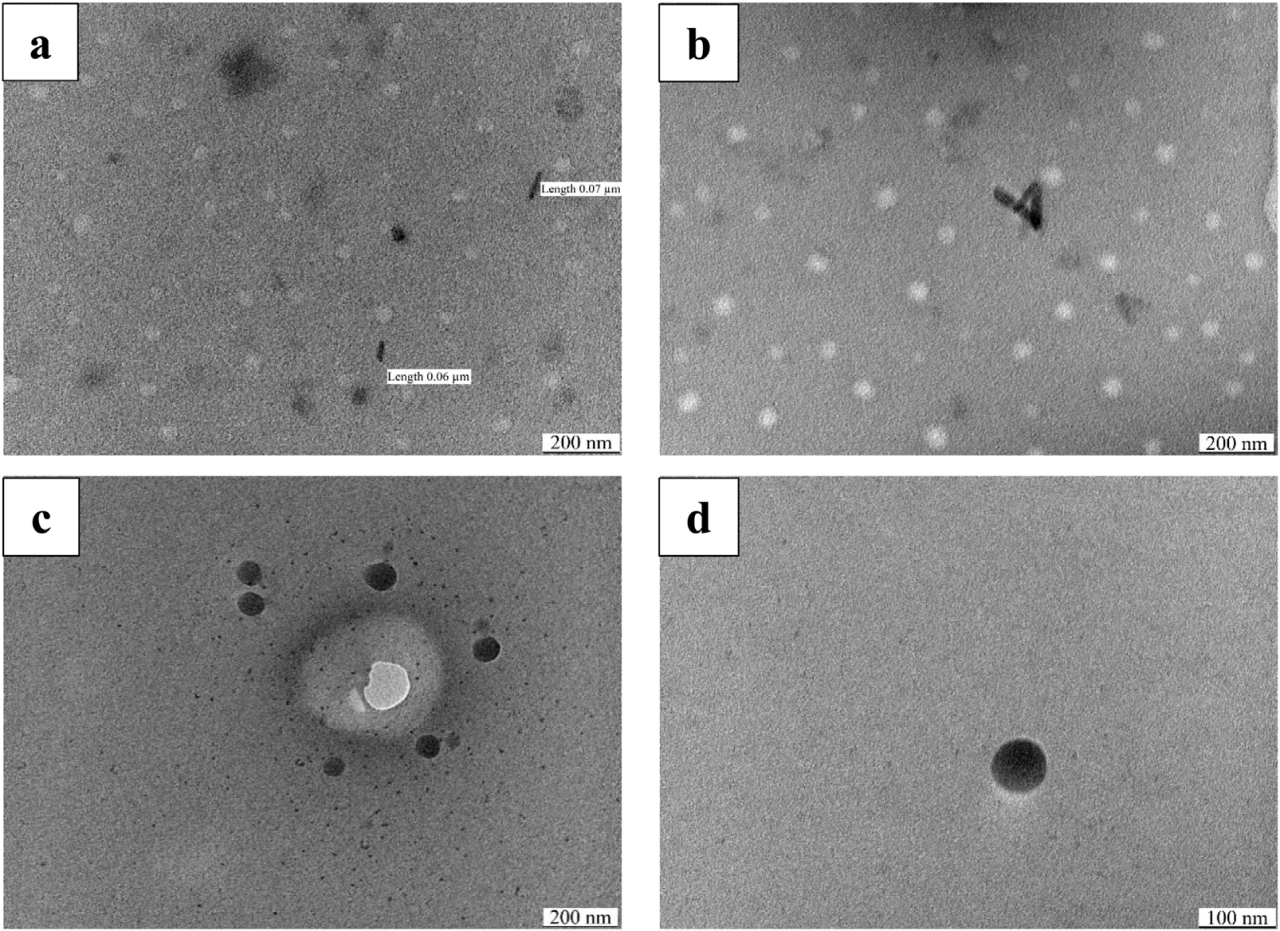
TEM images of Cr_2_O_3_NPs, both as-prepared **(a,b)** and calcined at 700 °C **(c,d)**.

Figures 4a–d demonstrate the transmission electron microscopy (TEM) images of Cr_2_O_3_ nanoparticles, both as-prepared and calcined at 700 °C. The images show that Cr_2_O_3_NPs mainly exhibit a spherical morphology, with some rod-shaped NPs also observed in the as-prepared sample. At a calcination temperature of 700 °C, Cr_2_O_3_NPs became agglomerated, which is in agreement with a previous work (Khalaji, 2021). It can be noted that the Cr_2_O_3_NP size determined by TEM ranged from ∼5 nm to 60 nm. The results show a strong similarity with the previous studies (Adnan and Mohammed, 2024; Mohammed et al., 2020). The biosynthesized NPs, in addition to the heat treatment, revealed more enhancements. According to the single phase, smaller crystalline size, and optical band gap, in addition to higher crystallinity, the study revealed the improvement of physiochemical characteristics better than those prepared through different methods in previous studies (Adnan and Mohammed, 2024; Ahmad et al., 2018; Hao et al., 2019; Khalaji, 2021; Ahmed Mohamed et al., 2020; Sackey et al., 2021). Furthermore, the observed enhancements of the achieved materials make them promising in photocatalytic applications (Khalaji and Pavel Machek, 2022).

### 3.2 Antibacterial activity of Cr_2_O_3_NPs

The Cr_2_O_3_NPs are known to possess strong antimicrobial activity and high inhibitory effects toward different microbes, such as *Escherichia coli*. Earlier studies have demonstrated the successful development of antimicrobials with a variety of nanoparticles, such as Zn, Ag, Cu, and TiO_2_ (Ali et al., 2021; Atta et al., 2021). The result obtained indicates that Cr_2_O_3_ in the nanoparticles enhances their antimicrobial activity. Additionally, during the reaction, superoxide radical (ROS) O_2_ is formed (Gyawali et al., 2024), which kills the microbes. This outcome is comparable to that of another study (Hasanin et al., 2023), which found that superoxide radical activity in the presence of Cr_2_O_3_NPs significantly disrupted microbial cell organelles such as the cell membrane, cytoplasmic fluid, and nucleic acids, resulting in cellular destruction.

**FIGURE 5:**
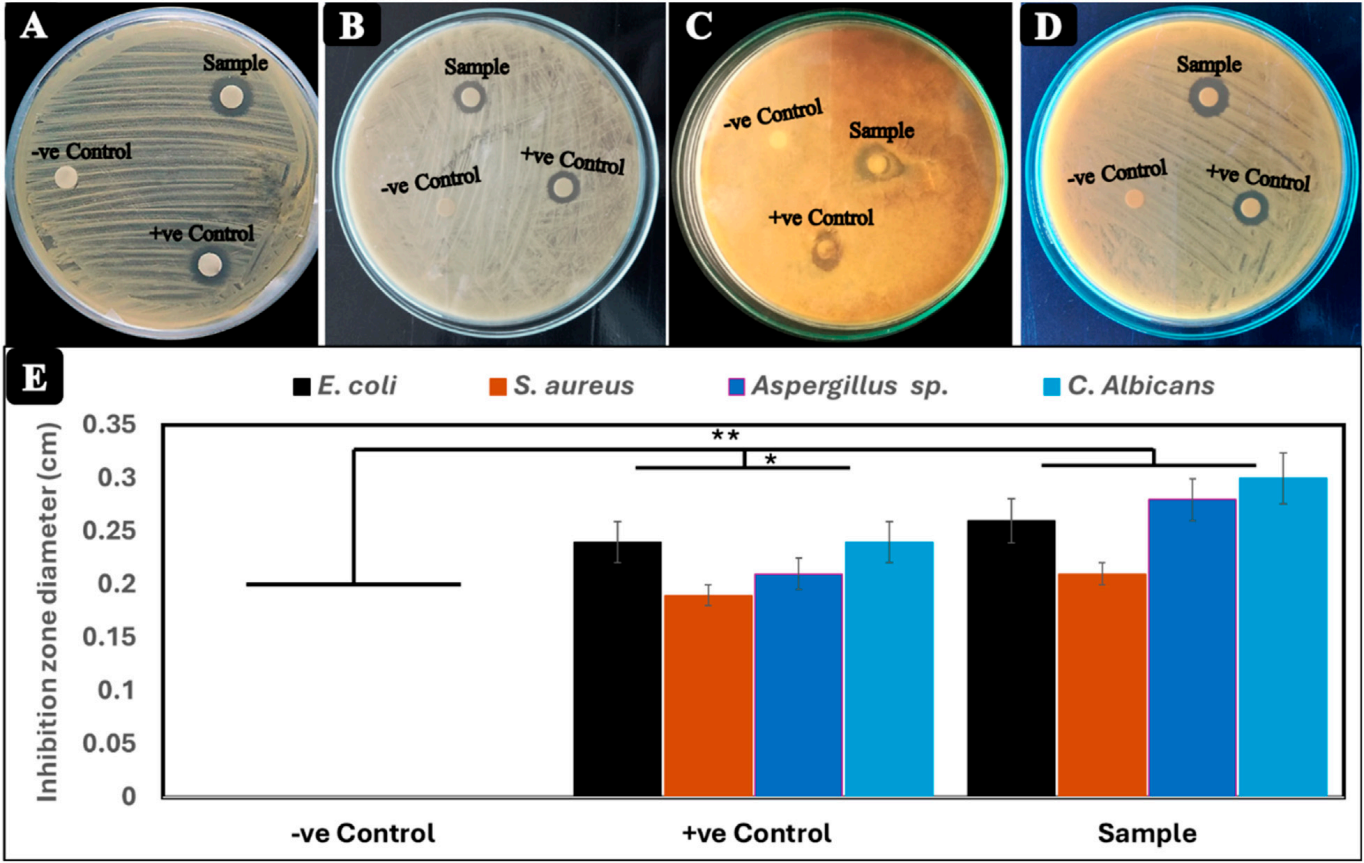
Antimicrobial activity of Cr_2_O_3_NPs film (test film), chromium nitrate (positive control), and filter paper with DMSO (negative control) against **(A)** *Escherichia coli*, **(B)** *Staphylococcus aureus*, **(C)** *Aspergillus* sp., and **(D)** *Candida albicans.* **(E)** Diameter (cm) of the inhibition zone.

In the present study, the antimicrobial activity of Cr_2_O_3_NPs was evaluated against four pathogens, including two bacterial strains, *Staphylococcus aureus* and *E. coli*, and two fungal species, *Candida albicans* and *Aspergillus sp*., along with the negative control (DMSO) and positive control (chromium nitrate), and the results are shown in Figure 5. The selected microbial strains for the determination of the antimicrobial activity of Cr_2_O_3_NPs are generally associated with human health. The antimicrobial activity evaluation through the disc diffusion method showed that the Cr_2_O_3_NPs discs produced inhibition zones of 0.26, 0.21, 0.28, and 0.3 cm for *E. coli*, *S. aureus, C. albicans*, and *Aspergillus* sp., respectively. These values are significantly better than the positive control (*p < 0.05) and higher than the negative control (**p < 0.01). In contrast, chromium nitrate (i.e., positive control) produced inhibition zones of 0.24, 0.19, 0.21, and 024 cm against the same set of microbial strains. On the other hand, filter paper with DMSO (i.e., negative control) did not produce any inhibition zones against the selected microbial strains, indicating that the antibacterial activity was exclusively attributed to the Cr_2_O_3_NPs (green synthesis), surpassing the antibacterial activity of the chemically synthetized form of Cr_2_O_3_NPs represented by chromium nitrate (as a positive control) (Dridi et al., 2018; Yasmeen et al., 2023).

Additionally, the study evaluated the effectiveness of Cr_2_O_3_NPs in inhibiting biofilm formation by persister cells from microbial isolates. The average inhibitory rate of Cr_2_O_3_NPs against microbial isolates was determined. Cr_2_O_3_NPs significantly reduced biofilm formation. Cr_2_O_3_NPs had a significant impact on biofilms formed by persister cells from all selected isolates (Al Marjani et al., 2021). Several factors influence the antibacterial mechanism of Cr_2_O_3_NPs, including the nanoparticles’ size and surface properties, the specific microbial strain, and the nanoparticles’ mode of action. Cr_2_O_3_NPs have the potential to induce oxidative stress in bacterial cells, leading to damage of DNA, proteins, and lipids. Reactive oxygen species (ROS), such as hydrogen peroxide (H_2_O_2_) and superoxide anion radicals ( $O_{2}^{-}$ ), can cause oxidative stress by interacting with and damaging cellular components (Benfield and Henriques, 2020; Rangayasami et al., 2020; Reygaert, 2018). The interaction of nanoparticles with the lipid bilayer of the bacterial membrane has the potential to change and disrupt the membrane structure. Figure 5 illustrates the mechanism of the antibacterial action of Cr_2_O_3_NPs.

### 3.3 Biocompatibility testing of Cr_2_O_3_NPs

**FIGURE 6:**
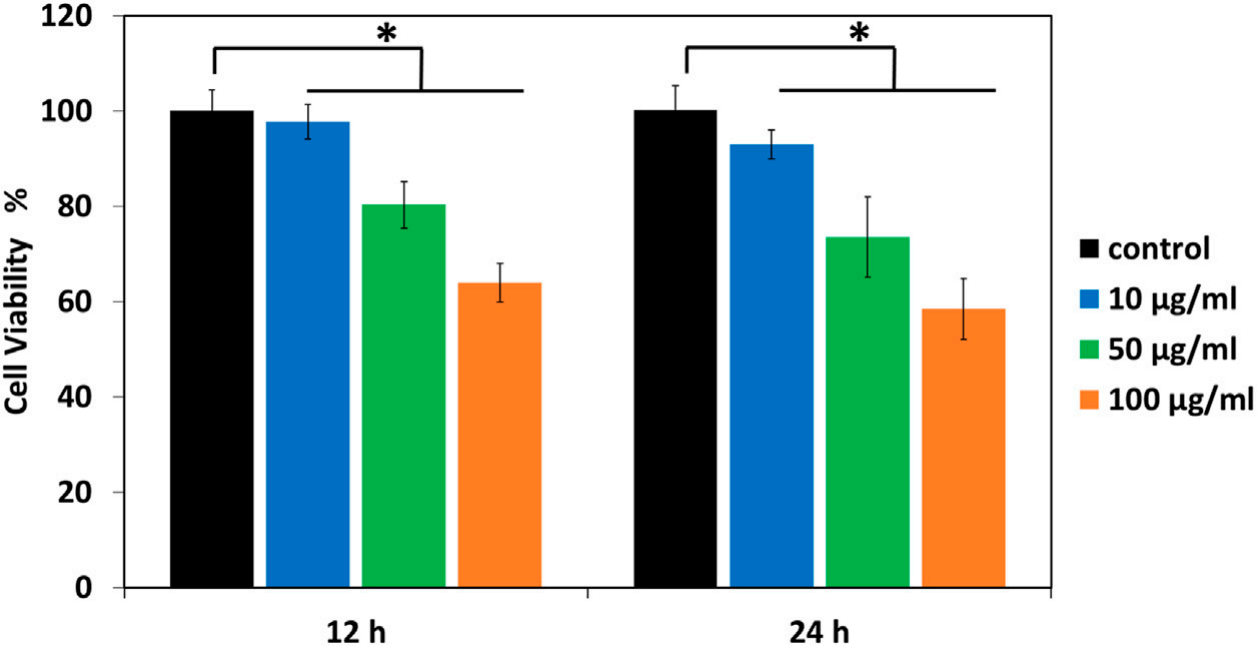
Viability of NIH-3T3 cells cultured in Cr_2_O_3_NPs in a 96-well plate. Absorption was recorded at 570 nm for all samples; *p < 0.05.

The synthesis of Cr_2_O_3_NPs for antimicrobial product development must ensure biocompatibility and non-toxicity. This study assessed the cytotoxicity of Cr_2_O_3_NPs on NIH-3T3 fibroblasts using the MTT assay, and the results are depicted in Figure 6. The viability of NIH-3T3 fibroblasts on Cr_2_O_3_NPs after 12 h was 98%, 80%, and 64% for concentrations of 10, 50, and 100 μg/mL, respectively, indicating no cytotoxic effect at low concentrations and low toxicity at high concentrations of the nanoparticles toward the cells (Figure 6). With continuous incubation for 24 h, viability gradually declines, reaching 93%, 74%, and 58% for Cr_2_O_3_NPs. The diminished cell viability may result from nutrient depletion in the growth medium. The results demonstrate satisfactory cell viability of Cr_2_O_3_NPs, with cell survival exceeding 90% relative to the control, indicating the acceptable cytotoxicity of Cr_2_O_3_NPs toward NIH-3T3 fibroblasts. The findings agree with a prior study indicating that Cr_2_O_3_NPs facilitated the proliferation of L929 cells while maintaining an acceptable toxicity threshold (Alarifi et al., 2016). Some studies indicated that chromium nitrate exhibits the highest toxicity, while others demonstrated that chromium synthesized through green methods has low toxicity (Hassan et al., 2019; Puerari et al., 2016; Sharma and Sharma, 2021; Sone et al., 2016). The current study’s findings indicate that Cr_2_O_3_NPs are predominantly non-toxic to mammalian cells, exhibiting toxicity only at elevated concentrations due to their diminutive size and volatile properties (Ahmed Mohamed et al., 2020). The present study does not permit the assessment of time-dependent cytotoxicity beyond the duration of 24 h. This represents a limitation of our study, which we will rectify in future research to deliver a more thorough evaluation of cytotoxicity over prolonged durations. Furthermore, we have elucidated the definitions of “low” and “high” concentrations in the manuscript. “Low concentrations” denote doses under 10 μg/mL, exhibiting no significant cytotoxicity, whereas “high concentrations” indicate levels exceeding this threshold, where cytotoxic effects are more evident.

## 4 Conclusion

In this study, Cr_2_O_3_NPs were successfully synthesized through a green method using the leaf extract of *Vicia monantha* Retz as both a reducing and capping agent. The synthesized Cr_2_O_3_NPs were thoroughly characterized using XRD, TEM, FT-IR spectroscopy, and UV–VIS spectroscopy. The green-synthesized Cr_2_O_3_NPs exhibited inhibitory activity against *Aspergillus sp*. and *Candida albicans*, with moderate effects observed against *S*. *aureus* and *E*. *coli*. Furthermore, the study suggests that Cr_2_O_3_NPs are predominantly non-toxic to mammalian cells, although some toxicity was noted under specific conditions. These findings indicate that green-synthesized Cr_2_O_3_NPs have potential applications in the medical field as effective antibacterial and antifungal agents. With further exploration, these nanoparticles could emerge as promising therapeutic agents in the future.

## Data Availability

The original contributions presented in the study are included in the article/supplementary material; further inquiries can be directed to the corresponding author.
